# Case Report: Midshaft clavicle fracture with concomitant high grade (Type V) acromioclavicular joint dislocation

**DOI:** 10.3389/fsurg.2022.885378

**Published:** 2022-08-09

**Authors:** Filip Cosic, Lukas Ernstbrunner, Greg A. Hoy, Keat S. Ooi, Eugene T. Ek

**Affiliations:** ^1^ Department of Orthopaedic Surgery, Austin Health, Heidelberg, VIC, Australia; ^2^ Melbourne Orthopaedic Group, Windsor, VIC, Australia

**Keywords:** clavicle, acromioclavicular (AC) joint, internal fixation, joint stabilization, fracture

## Abstract

**Introduction:**

Concomitant acromioclavicular joint dislocation and midshaft clavicle fracture are rare injuries, generally resulting from high energy trauma, with limited previous experience in management.

**Case:**

A 30 year old male presented following a pushbike accident. He had suffered a head on collision with another cyclist. Radiographic examination demonstrated a displaced midshaft clavicle fracture with a Rockwood Type V acromioclavicular joint dislocation. Operative management was undertaken using a dual plating technique. At six month follow up the patient demonstrated full range of motion and had no pain.

**Conclusion:**

Appropriate radiographic evaluation and careful intraoperative assessment are required using the principles of management for acromioclavicular joint injuries, along with stabilization of the mid-clavicular fracture to reduce the risk of non-union.

## Introduction

Clavicle fractures and acromioclavicular joint dislocations are common orthopaedic injuries, and frequently result from a fall on to the tip of the shoulder (1). However, only a handful of cases have been reported in the literature of concomitant acromioclavicular joint dislocation and midshaft clavicle fracture, which generally result from high energy injuries with force directed to the lateral shoulder, including motor vehicle crashes, falls from horseback and falls from pushbikes (2–8). Here we report a case of a cyclist presenting with this injury following a fall from a pushbike, which was successfully managed surgically. Through this case we illustrate the technique of surgical management of this injury pattern with a dual plating and discuss the current literature.

## Case description

**Figure 1:**
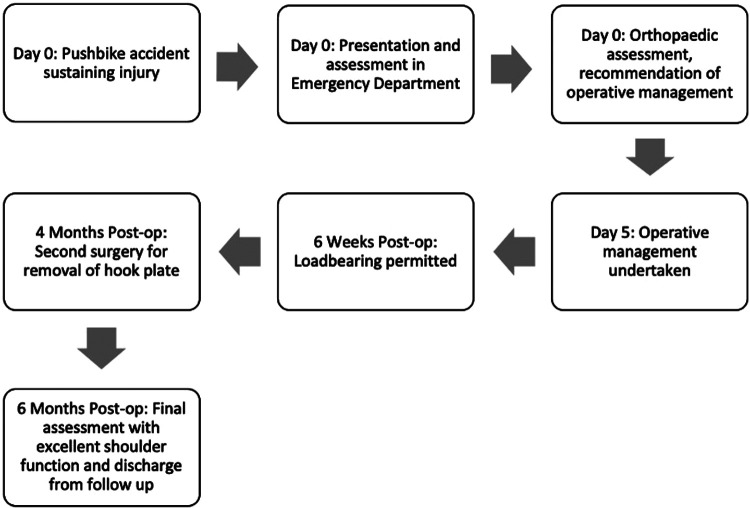
Timeline of presentation and management.

**Figure 2:**
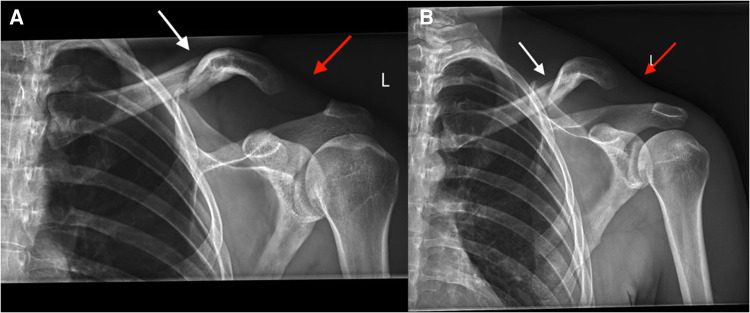
Radiographs demonstrating midshaft clavicle fracture with associated acromioclavicular joint dislocation (white arrow demonstrating the fracture site, red arrow demonstrating the dislocation site).

**Figure 3:**
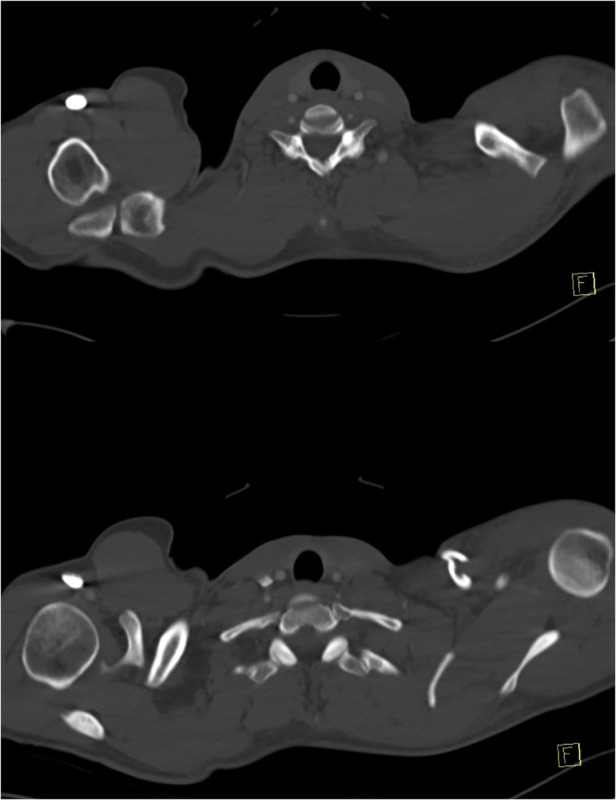
Axial computed tomography demonstrating concomitant midshaft clavicle fracture and Rockwood Type V acromioclavicular joint dislocation.

A 30 year old right hand dominant male presented to our institution following a pushbike accident (Figure 1). He had suffered a head on collision with another cyclist, both travelling at approximately 30 km/h and was thrown from his bicycle. Examination revealed deformity of the shoulder girdle and abrasion over the posterior aspect of the shoulder. The upper limb was neurovascularly intact. The patient had a past medical history of ankylosing spondylitis and IgA nephropathy. Radiographic examination of the right clavicle demonstrated a displaced midshaft clavicle fracture with a Rockwood Type V acromioclavicular joint dislocation (Figure 2) (9). The injury was confirmed on computed tomography (CT) scan undertaken as part of the routine trauma protocol (Figure 3). Operative management of the injury was recommended and this was undertaken as outpatient treatment five days following the initial injury.

**Figure 4:**
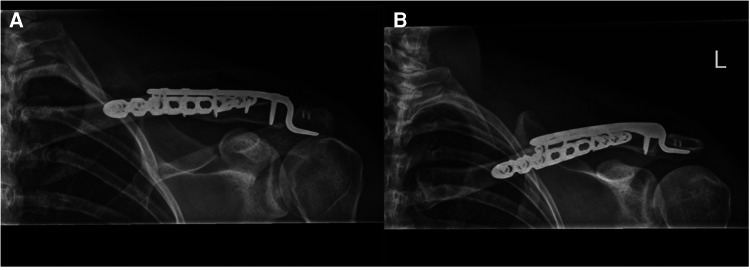
Radiographs demonstrating fixation of the fracture and dislocation with a hook plate and anterior clavicle plate.

Operative management was performed with the patient placed in beach chair position, with the head inclined approximately 30 degrees. A transverse incision was made over the clavicle and extended laterally over the AC joint. The lateral clavicle was found to be posteriorly translated with complete disruption of the acromioclavicular joint capsule and ligaments, and coracoclavicular ligaments. The fracture fragments were identified and reduced with bone reduction forceps. The reduction was held with a lag screw applied using standard AO techniques. The acromioclavicular joint was then reduced and a precontoured 3.5 mm hook plate (Synthes, West Chester, PA, USA) applied to hold the AC joint reduction. This reduction and fixation was then augmented with a precontoured mid shaft clavicle locking compression plate (Synthes, West Chester, PA, USA) applied to the anterior surface of the clavicle bridging the fracture site. This provided a strong orthogonal construct stabilizing both the clavicle and the AC joint complex. Suture anchor repair of the acromioclavicular ligaments was then undertaken to augment the repair of the AC joint complex (Figure 4).

The post-operative protocol consisted of no loadbearing for six weeks, the use of a broad arm sling for comfort and range of motion exercises as tolerated with the only range restriction being no shoulder abduction past 90 degrees. Hook plate removal was undertaken four months following initially surgery.

**Figure 5.**
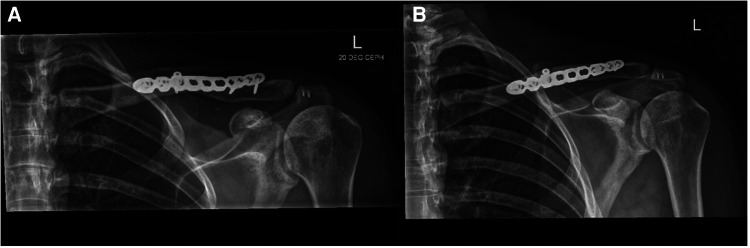
Radiographs demonstrating a united fracture and stable acromioclavicular joint post removal of hook plate.

At six month follow up the patient demonstrated full range of motion and had no pain. Radiographs demonstrated a united fracture and stable acromioclavicular joint (Figure 5). The patient had an American Shoulder and Elbow Surgeons Shoulder Score of 90 (out of 100) at final follow up.

## Discussion

Clavicular fractures and acromioclavicular joint dislocations as isolated injuries are common injuries (1). When these injuries occur concurrently it is typically associated with a distal clavicle fracture (10). The combination of midshaft clavicle fracture with an acromioclavicular joint dislocation is rare, with only a handful of previously reported cases in the literature (2–8).

Previous case reports have described this injury in association with high energy trauma, notably motor vehicle and horse riding accidents. Psarakis et al. described a Type V acromioclavicular joint injury in association with a midshaft clavicle fracture following a motor vehicle accident which was successfully managed with a precontoured locking plate fracture fixation and a suture button reconstruction of the coracoclavicular ligaments (2). The patient returned to full activity and had full range of shoulder motion at 18 months post-operatively. Yeh et al. described a Type IV acromioclavicular joint injury and midshaft clavicle fracture following a fall from a horse managed successfully with a locking plate and semitendinosus allograft reconstruction of the coracoclavicular ligaments (5). The patient returned to pre-injury activity and demonstrated painless full range of motion at one year post-operatively.

In this present case, the fracture/dislocation was successfully managed with a dual plating technique with anterior locking plate fixation of the clavicular fracture and hook plate fixation of the acromioclavicular joint injury, a technique that to the authors' knowledge has not been previously used. This fixation technique allows for a rigid construct and stabilisation of the fracture, allowing early mobilisation. A dual plating technique also reduces the stress applied across the clavicle fracture site by a concomitant coracoclavicular reconstruction potentially promoting union of the clavicular fracture. This case demonstrates successful union of the clavicular fracture using this technique along with a stable AC joint upon removal of the hook plate. Other management options previously described across several decades for this rare injury have included fracture plating with variations of coracoclavicular ligament reconstruction, and have also included isolated surgical management of the coracoclavicular injury with non-operative management of the clavicle fracture, along with non-operative management of both injuries (2–8).

In the diagnosis of these injuries the authors recommend careful preoperative evaluation of radiographs. Where there is suspicion for a concomitant midshaft clavicle fracture and acromioclavicular joint dislocation bilateral dedicated acromioclavicular joint radiographs should be performed with a low threshold for computed tomography. The authors advocate for operative management of this injury due to the increased instability generated by the segmental nature of the injury pattern, particularly in light of current evidence suggesting a higher rate of non-union of clavicle fractures than previously described (11). Regardless of the operative management undertaken these injuries present a surgical challenge and require careful pre-operative planning to ensure appropriate intraoperative management, implant availability and to mitigate the risk of failure of fixation and subsequent reoperation.

This case demonstrates successful management of a rare combined injury with a dual plating technique. Appropriate radiographic evaluation and careful intraoperative assessment are required using the principles of management for acromioclavicular joint injuries, along with stabilization of the mid-clavicular fracture to reduce the risk of non-union.

## Data Availability

The original contributions presented in the study are included in the article/Suplementary Material, further inquiries can be directed to the corresponding author/s.
